# Physicochemical Properties of Hematite Nanoparticles Obtained via Thermogravimetric Conversion of Biosynthesized Nanomaghemite

**DOI:** 10.3390/ma18204677

**Published:** 2025-10-12

**Authors:** Juan A. Ramos-Guivar, Mercedes del Pilar Marcos-Carrillo, Renzo Rueda-Vellasmin, Erich V. Manrique-Castillo, Noemi-Raquel Checca-Huaman, Bruno L. D. Santos, Waldemar A. A. Macedo, Edson C. Passamani

**Affiliations:** 1 Grupo de Investigación de Nanotecnología Aplicada para Biorremediación Ambiental, Energía, Biomedicina y Agricultura (NANOTECH), Facultad de Ciencias Físicas, Universidad Nacional Mayor de San Marcos, Av. Venezuela Cdra 34 S/N, Ciudad Universitária, Lima 15081, Peru; 10130030@unmsm.edu.pe (M.d.P.M.-C.); emanriquec@unmsm.edu.pe (E.V.M.-C.); 2 Centro Brasileiro de Pesquisas Físicas (CBPF), R. Xavier Sigaud, 150, Urca 22290-180, RJ, Brazil; nomifsc@cbpf.br; 3 Centro de Desenvolvimento da Tecnologia Nuclear, CDTN, Belo Horizonte 31270-901, MG, Brazil; bruno.santos@cdtn.br (B.L.D.S.); wmacedo@cdtn.br (W.A.A.M.); 4 Programa de Pós-Graduação em Ciências-Física de Materiais, Universidade Federal de Ouro Preto-UFOP, Ouro Preto 35402-136, MG, Brazil; 5 Department of Physics, Federal University of Espírito Santo-UFES, Vitória 29075-910, ES, Brazil

**Keywords:** hematite nanoparticles, nanomaghemite, thermogravimetric conversion, Mössbauer spectroscopy, magnetic properties, biosynthesis

## Abstract

Hematite nanoparticles ($\alpha -{\mathrm{Fe}}_{2}O_{3}\;\mathrm{NPs}$) were synthesized through a thermal conversion of synthetic and biosynthesized nanomaghemite ($\gamma -{\mathrm{Fe}}_{2}O_{3}\;\mathrm{NPs}$) precursors. X-ray diffraction data confirmed phase-pure hematite with crystallite sizes of 54 and 56 nm for the H1 and H2 samples, respectively. Transmission electron microscopy (TEM) revealed a bimodal-like distribution feature (peaks at 18.5 and 35.5 nm) for the H1 sample, while the histogram plot of the H2 sample displayed a homogeneous particle size distribution with a mean size of 28 nm. X-ray photoelectron spectroscopy confirmed Fe^3+^ ions as the dominant oxidation state in both samples. In addition, while ^57^Mössbauer spectroscopy indicated relaxation effects and line broadening for the H1 sample at both 300 K and 16 K, consistent with incomplete γ→α transformation, the H2 sample exhibited spectra at the same temperatures resembling a bulk-like hematite. Magnetometry supported these findings since the H1 sample showed enhanced coercivity (2.2 kOe) and remanence (0.23 emu/g), features attributed to a residual ferrimagnetic contribution of $\gamma -{\mathrm{Fe}}_{2}O_{3}\;\mathrm{NPs}$, and the H2 sample exhibited weaker ferromagnetism, as typically found in nanoscale hematite. These results highlight the synergistic use of X-ray photoelectron and Mössbauer spectroscopies, and magnetic measurements to reveal subtle multiphase coexistence, demonstrating that precursor chemistry and biosynthetic functionalization decisively govern the structural and magnetic evolution of $\gamma \to \alpha -{\mathrm{Fe}}_{2}O_{3}\;\mathrm{NPs}$.

## 1. Introduction

Iron oxides are among the most studied metal oxides due to their abundant availability, low toxicity, and versatile physicochemical properties [1,2]. Among these, hematite (α-Fe_2_O_3_) is the most thermodynamically stable phase under ambient conditions and exhibits unique structural, electronic, and magnetic characteristics that make it attractive for a broad range of applications [3]. α-Fe_2_O_3_ crystallizes in the rhombohedral corundum structure (space group R-3c), where Fe^3+^ cations occupy two-thirds of the octahedral sites within a hexagonal close-packed array of oxygen anions [4]. This arrangement confers high chemical stability and a moderate bandgap energy of ~2.1 eV, making it suitable for photoelectrochemical water splitting [5], photocatalysis [6], lithium-ion batteries, and environmental remediation [7], among other applications.

When the particle size is reduced to the nanometric scale (< 100 nm), α-Fe_2_O_3_ exhibits remarkable size-dependent properties. Nanohematite ($\alpha -{\mathrm{Fe}}_{2}O_{3}\;\mathrm{NPs}$) shows a substantial increase in surface area, modified electronic structure, and changes in magnetic behavior due to finite-size effects and spin disorder at the particle surface [8]. While bulk α-Fe_2_O_3_ is antiferromagnetic below the Morin transition temperature (~260 K) [9] and weakly ferromagnetic above this point due to spin canting, $\alpha -{\mathrm{Fe}}_{2}O_{3}\;\mathrm{NPs}$ often displays superparamagnetic or weak ferromagnetic characteristics at room temperature (RT) [10]. These size-dependent features broaden the potential applications of nanohematite [2,10,11]. In other words, the increased surface area and modified electronic structure enhance its catalytic activity [10], while the tunable magnetic behavior underpins its use in targeted drug delivery [2] and magnetic hyperthermia [11]. In addition, it should be mentioned that such applications are particularly effective when the particles are uniform and highly dispersed.

Various synthetic strategies have been employed to produce $\alpha -{\mathrm{Fe}}_{2}O_{3}\;\mathrm{NPs}$, including sol–gel processing, hydrothermal and solvothermal methods, precipitation, and thermal decomposition [12,13,14,15]. Recently, biosynthetic approaches have attracted considerable attention as sustainable and environmentally benign alternatives. Such methods exploit plant extracts, microorganisms, or biopolymers as reducing and stabilizing agents, minimizing the use of hazardous chemicals [16]. However, obtaining phase-pure $\alpha -{\mathrm{Fe}}_{2}O_{3}\;\mathrm{NPs}$, with controlled size, morphology, and magnetic properties, remains a challenge. Frequently, the initial product consists of intermediate iron-oxide phases, such as maghemite (γ-Fe_2_O_3_) or magnetite (Fe_3_O_4_), which must be transformed into α-Fe_2_O_3_ through thermal treatments at temperatures above 873 K [4].

Thermogravimetric analysis (TGA) is widely used to monitor the γ→α transformation [17], as it provides insights into the weight loss associated with dehydration, oxidation, and crystallization steps during the heating process of samples. The conversion of biosynthesized nanomaghemite to nanohematite ($\gamma \to \alpha -{\mathrm{Fe}}_{2}O_{3}\;\mathrm{NPs}$), via controlled thermal treatments, is of particular interest, as it allows phase engineering while preserving nanometric features. Nonetheless, key challenges remain, including particle growth during calcination, aggregation, and the precise control of magnetic properties through size and morphology regulation.

In a previous work [18], Marcos-Carrillo et al. developed an eco-friendly synthesis route for iron-oxide nanoparticles (IONPs) using *Chenopodium quinoa* extract as a reducing and stabilizing agent. By varying extract concentrations from 5 to 50% (*w*/*v*), they obtained nanoparticles with mean crystallite sizes of ~7–8 nm, where the phase composition shifted from mixed γ-Fe_2_O_3_–goethite to predominantly γ-Fe_2_O_3_ at higher loadings. Thermogravimetric analysis confirmed increasing organic content with extract concentration, while FTIR spectra evidenced surface functionalization with carboxyl and amide groups originating from flavonoids and proteins in the extract. The particles exhibited hard ferrimagnetic behavior, with saturation magnetizations of ~42 emu/g and ~11 emu/g depending on extract content. Zeta potential measurements showed stable dispersions above neutral pH, with the point of zero charge decreasing from ~6.3 to ~3.8 as the extract concentration increased, reflecting enhanced surface hydroxylation. Thus, these observed structural, magnetic, and colloidal properties underline the potential of quinoa-derived IONPs for environmental applications, particularly in remediation processes requiring magnetic recovery and surface-mediated interactions.

In this work, we report the synthesis of $\alpha -{\mathrm{Fe}}_{2}O_{3}\;\mathrm{NPs}$ from biosynthesized γ-Fe_2_O_3_ precursors, followed by a thermal transformation under controlled conditions, using thermogravimetric equipment. The structural and magnetic properties of the resulting $\alpha -{\mathrm{Fe}}_{2}O_{3}\;\mathrm{NPs}$ were systematically characterized to elucidate the impact of thermal treatment parameters on phase purity, crystallite size, and magnetic behavior. This study provides valuable insights into the optimization of sustainable routes for producing nanohematite with tailored properties for advanced applications in catalysis, energy conversion, and magnetic-based technologies.

## 2. Materials and Methods

### 2.1. α-Fe_2_O_3_ Preparation Using the Shimadzu Thermogravimetry Equipment

**Scheme 1:**
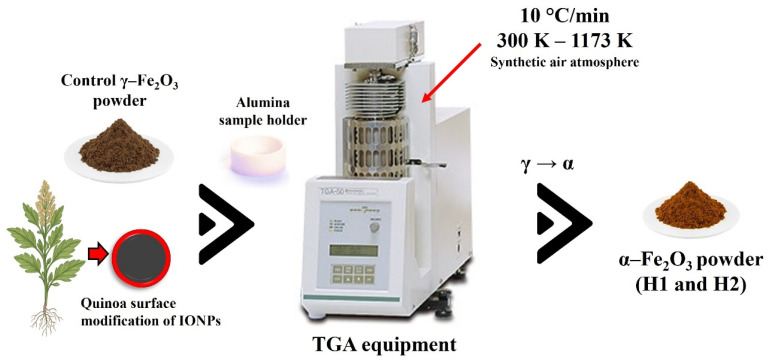
Thermal transformation of γ–Fe_2_O_3_ → α–Fe_2_O_3_ using TGA equipment.

The $\gamma \to \alpha -{\mathrm{Fe}}_{2}O_{3}$ transformation was conducted through a controlled calcination process using a thermogravimetric analyzer (Shimadzu TGA-50, Shimadzu, Kyoto, Japan). Prior to this, preliminary TGA was performed to evaluate the thermal stability and decomposition profile of the samples, guiding the selection of the final calcination temperature, see Scheme 1. An amount of 16 mg of γ-Fe_2_O_3_ was placed in the alumina sample-holder and subjected to heating in a synthetic air atmosphere, starting from 300 K and increasing at a rate of 10 °C/min until reaching a maximum temperature of 1173 K. This procedure was repeated as many times as necessary to obtain a sufficient amount of material for comprehensive characterization and confirmation of the α-phase through complementary techniques. This maximum temperature was selected as indicated in the literature, which reports that the thermal transition from γ-Fe_2_O_3_ to α-Fe_2_O_3_ typically begins above 873 K and becomes predominant around 1073–1173 K [4,19]. Two types of α-Fe_2_O_3_ samples were processed. The first (H1) consisted of calcined 13.8 nm γ-Fe_2_O_3_ NPs (control sample). The second sample (H2) consisted of 7.0 nm γ-Fe_2_O_3_ NPs, previously functionalized with quinoa organic elements (QE10 sample) [18], aiming to investigate how the organic covering layer (shell) influences the thermal stability behavior and phase transformation pathway.

For the H1 sample, the total initial mass was 104.63 mg, and the final mass after calcination was 101.50 mg, corresponding to an average mass loss of approximately 2.75%.

In contrast, the H2 sample had an initial mass of 94.21 mg and a final mass of 68.45 mg, reflecting a significantly higher mass loss of about 27%, suggesting a high weight loss related to the quinoa organic covering layer.

### 2.2. Characterization

Structural properties of the iron-oxide nanopowder samples were analyzed using a RIGAKU Ultima IV diffractometer (Rigaku, Tokyo, Japan), equipped with conventional CuK_α_ radiation (λ = 1.5418 Å), and operated in Bragg–Brentano geometry. The X-ray diffraction (XRD) measurements were carried out over a 2*θ* range of 20° to 80°, with a step size of 0.02° and a counting time of 2 s per step. These experimental conditions were applied to both H1 and H2 samples.

The morphological characteristics of the samples were acquired using a JEOL 2100F transmission electron microscope (JEOL, Tokyo, Japan), operating at 200 kV in transmission (TEM) mode. TEM samples were prepared by dispersing the solid powder in an organic solvent. Approximately 20 mg of the material was placed in a 1.5 mL Eppendorf tube and suspended in 1 mL of acetone. The suspension was sonicated in an ultrasonic bath for 5 min to break up agglomerates and obtain a stable NP`s dispersion. After sonication, 5 µL of the supernatant was carefully withdrawn using a micropipette and deposited onto a TEM grid (Lacey carbon film, 300 mesh copper support, grid hole size ~63 µm, Ted Pella, Inc., Redding, CA, USA). The grids were then dried under ambient conditions prior to imaging. The particle size distribution (PSD) histograms were determined by counting between 800 and 1000 particles from 30 to 35 photographs utilizing Image J software version 1.54g. The histograms were modeled using a log-normal distribution [20]. Polydispersity values were ascertained by calculating the standard deviation of the log-normal distribution.

Fourier Transform Infrared (FTIR) spectra were recorded using an Agilent Cary 630 spectrometer (Agilent Technologies, Santa Clara, CA, USA) equipped with an ATR module. The spectra were acquired in the 4000–650 cm^−1^ range, with a spectral resolution of 4 cm^−1^ and a total of 16 scans per sample, expressed in transmittance mode. Although ATR does not measure true transmission spectra, the data are presented in pseudo-transmittance coordinates (%T vs. wavenumber), as provided by the instrument software, in order to maintain consistency with previous studies and facilitate spectral comparison.

X-ray photoelectron spectroscopy (XPS) measurements were obtained in an ultra-high vacuum system (SPECS GmbH, Berlin, Germany) equipped with a PHOIBOS 150-MCD electron analyzer and an Al-Kα X-ray source (1486.7 eV, 15 kV, 300 W), using a pass energy of 50 eV and a step size of 0.5 eV for the wide spectra, and 20 eV/0.1 eV for the high-resolution XPS spectra. Charge compensation was obtained by using a flood gun (20 µA emission/2 eV energy). The high-resolution XPS spectra were fitted with Gaussian-Lorentzian mixed functions for Fe 2p, O 1s, and C 1s peaks. The Fe 2p doublets were fitted constraining the integrated area ratios of the two components to the expected values (2p_3/2_/2p_1/2_ = 2) [21]. The data processing and the fittings were performed using the CasaXPS software 2.3.26, and all XPS spectra were plotted using OriginPro software v.19. The residual standard deviation (STD) was kept below 1.5 over the background residual STD to ensure a good fit of experimental data.

The ^57^Fe Mössbauer spectra of the samples were acquired in transmission mode using a Lake Shore CCS-800-205 closed-cycle helium cryostat (Lake Shore Cryotronics, Westerville, OH, USA), allowing measurements at 300 K and 16 K. A ^57^Co(Rh) source (25 mCi) maintained at 300 K was employed, while the absorbers were measured either at room temperature or under cryogenic conditions. The Doppler velocity (Doppler shifting) was generated by moving the ^57^Co(Rh) source in a sinusoidal mode in ten of mm/s range (512 channels, unfolded). The calibration of the velocity scale (energy scale) was performed with an α-Fe reference foil (12.7 μm thick) at 300 K. The absorbers (powdered samples) were mounted in nylon sample holders with effective thicknesses corresponding to ~0.1 mg of ^57^Fe per cm^2^. To minimize external disturbances, both the spectrometer and the sample holder were mounted on an anti-vibration platform.

Magnetization versus field, *M*(*H*), measurements were carried out in a Physical Property Measurement System (PPMS, Evercool II, Quantum Design Inc., San Diego, CA, USA) equipped with the Vibrating Sample Magnetometer (VSM) option. The experiments were conducted under applied magnetic fields up to ±50 kOe, with data collected at both room temperature (300 K) and low temperature (5 K).

## 3. Results and Discussions

### 3.1. TGA and α-Fe_2_O_3_ Crystallization

**Figure 1:**
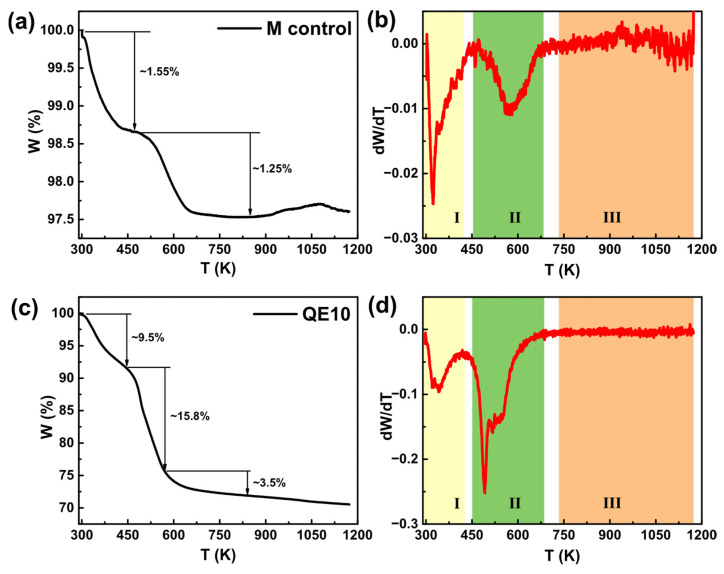
TG profiles showing the thermal decomposition of the M control (H1) and QE10 (H2) samples. (**a**,**b**) Thermal behavior to form H1, showing volatile release, molecule desorption, and phase transformation to α-Fe_2_O_3_. (**c**,**d**) Thermal decomposition to form H2, highlighting mass losses associated with volatile compounds and organic decomposition, and subsequent α-Fe_2_O_3_ stabilization. Shaded regions indicate different thermal events.

TGA and derivative thermogravimetric (DTG) analyses of the M (H1 at the end) and QE10 (H2 at the end) samples reveal distinct thermal decomposition behaviors, as seen in Figure 1a–d. For the M control, three regions are identified: Region-I (300–450 K), a minor mass loss (~1.55%) is associated with the release of volatile species intrinsic to γ-Fe_2_O_3_; Region-II (450–700 K), a smaller loss (~1.25%) corresponds to the removal of more thermally resistant molecules; Region-III (700–1173 K), a slight mass gain is observed, attributed to oxygen incorporation during the γ-Fe_2_O_3_-to- α-Fe_2_O_3_ transformation [4]. The QE10 (H2) sample also exhibits three regions, but now with greater mass losses due to its organic functionalization with quinoa. Thus, the regions can be identified as: Region-I (300–400 K), the evaporation of liquids and volatile compounds results in a ~9.5% loss; Region-II (400–700 K), a pronounced loss (~15.8%) occurs, corresponding to the decomposition of organic groups; and Region-III (700–1173 K), a mass stabilization is observed, indicating the formation of α-Fe_2_O_3_ without additional oxygen uptake. More important, the mass gain observed for the resulting H1 sample is absent in the H2 sample, which could reflect the structural rearrangement and oxygen accommodation, characteristic of α-Fe_2_O_3_ crystallization [22,23].

### 3.2. X-Ray Diffraction and Rietveld Analysis

**Figure 2:**
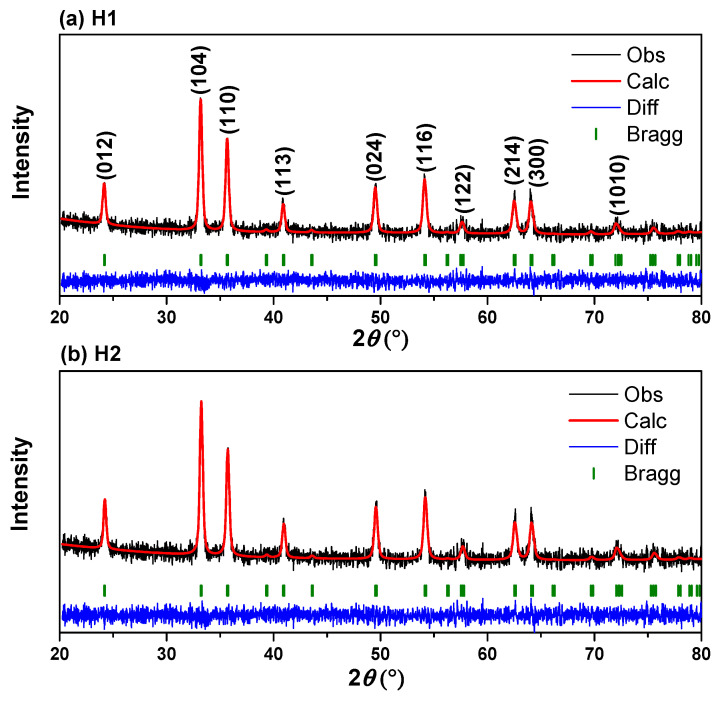
Refined X-ray diffractograms of the (**a**) H1 and (**b**) H2 samples. The black lines indicate the observed diffractograms, the red lines the calculated patterns, and the horizontal blue line refers to the residual profiles; the green vertical lines are related to the Bragg peaks. The Miller planes are indexed at the top of each diffraction peak in (**a**).

Figure 2a,b shows the refined X-ray diffractograms for the H1 and H2 samples. The main peaks assigned to the trigonal α-Fe_2_O_3_ phase were observed. No additional Bragg peaks, which could be related to spurious/secondary phases, were detected in both X-ray diffractograms. The mean crystallite sizes were found to be ca. 54 nm and 56 nm for the H1 and H2 samples, respectively. These values for the crystallites indicate that the final samples (H1 and H2) are still in nanometric scale, as also found for the precursor samples (before the heating). Table 1 shows the refined crystallographic parameters for both H1 and H2 samples.

The interplanar distances were calculated using the Equation (1), considering a trigonal crystal symmetry (hexagonal axes), and the results for characteristic Miller planes are summarized in Table 2.

(1) $$\frac{1}{d^{2}}=\frac{4}{3}\left(\frac{h^{2}+hk+k^{2}}{a^{2}}\right)+\frac{l^{2}}{c^{2}}$$

### 3.3. TEM Analysis

**Figure 3:**
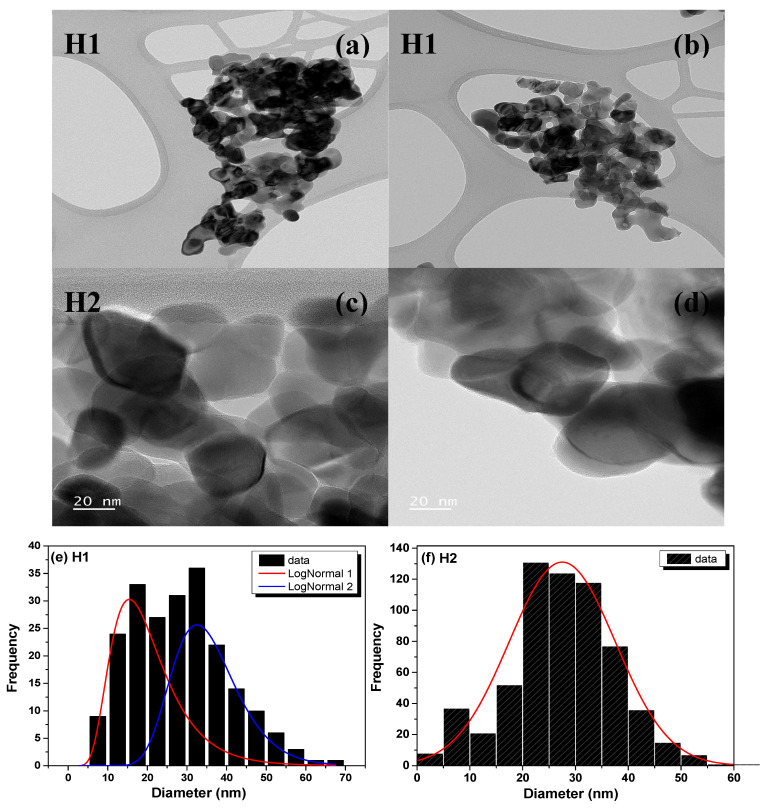
Representative TEM images for the H1 and H2 samples (**a**–**d**) and their corresponding PSD histograms (**e**,**f**). In (**a**,**b**) the bar scale is 100 nm.

Figure 3a–d displays the representative images for the H1 and H2 samples. In Figure 3e, the PSD histogram reveals mean particle sizes of 18.5(1) nm and 34.5(1) nm for the H1 sample. Therefore, a bimodal distribution was observed for this sample, probably associated with residual γ-Fe_2_O_3_ NPs either coated by α-Fe_2_O_3_ NPs (core–shell feature) or in between α-Fe_2_O_3_ NPs (the apparent absence of the γ-Fe_2_O_3_ signal in the XRD can be explained considering their small sizes and quantity compared to the well-crystallized α-Fe_2_O_3_ NPs that have larger sizes). In contrast, a single distribution with an average size of 28(1) nm was found for the H2 sample, as illustrated in Figure 3f.

The crystallite sizes of the α-Fe_2_O_3_ NPs, estimated from X-ray diffraction using the Scherrer equation, were 54 nm for the H1 sample and 56 nm for the H2 sample. These values are consistently larger than the particle sizes obtained from TEM analysis, which yielded mean diameters of 18.5 nm and 34.5 nm for H1 (showing a bimodal distribution), and 28 nm for H2. This discrepancy is frequently reported in the literature and arises because X-ray diffraction and TEM probe different physical quantities. X-ray diffraction, through the Scherrer equation, provides the mean size of coherent diffracting domains, while TEM directly images the external morphology of individual NPs [24,25]. Consequently, X-ray diffraction can yield larger values when particles contain multiple crystallites or extended crystalline domains, whereas TEM reflects the particle size observed in a limited field of view.

For the H1 sample, the coexistence of two particle populations (18.5 and 34.5 nm) suggests heterogeneous growth. The smaller particles observed by TEM images may still be part of larger coherent domains detected by X-ray diffraction, explaining the higher Scherrer’s sizes. In contrast, for the H2 sample, the TEM mean value of 28 nm is closer to twice the value obtained from the X-ray experiment (56 nm), consistent with a narrower PSD and fewer subunits per crystallite. Similar differences between TEM and X-ray diffraction derived sizes have been reported for hematite and other oxides, where instrumental broadening corrections, strain effects, and the choice of Scherrer constant strongly influence the calculated values [24,26,27]. Additionally, the limited sampling in TEM experiments compared to the bulk-averaging nature of X-ray diffraction can further accentuate these discrepancies [25]. Overall, the combined TEM and X-ray analyses indicate that the α-Fe_2_O_3_ NPs are nanocrystalline, with coherent domains of 50–60 nm, but consisting of smaller morphological units, consistent with partial aggregation and polycrystalline organization.

The TEM findings (H1 bimodal, H2 unimodal) are central to explaining apparent divergent magnetic and Mössbauer behaviors in this ensemble of α-Fe_2_O_3_ NPs, as will be shown in the next sections. However, at this stage, we would like to point out that, for example, Tadić et al. [28] have shown that α-Fe_2_O_3_ NPs with dual-size populations generate mixed magnetic dynamics: smaller particles are dominated by spin relaxation and/or superparamagnetic behavior, while larger particles usually retain coercivity and are found in magnetically blocked state. Similarly, size-dependent structural defect studies (7–120 nm) has also shown that smaller α-Fe_2_O_3_ NPs retain higher γ-Fe_2_O_3_-like surface defects and lattice disorder [29], which produce residual ferrimagnetic signatures. In general, it can be said that these results are consistent with those found in our structural and morphological studies and will also be supported by Mössbauer and magnetic data to be discussed ahead.

### 3.4. ATR-FTIR Analysis

**Figure 4:**
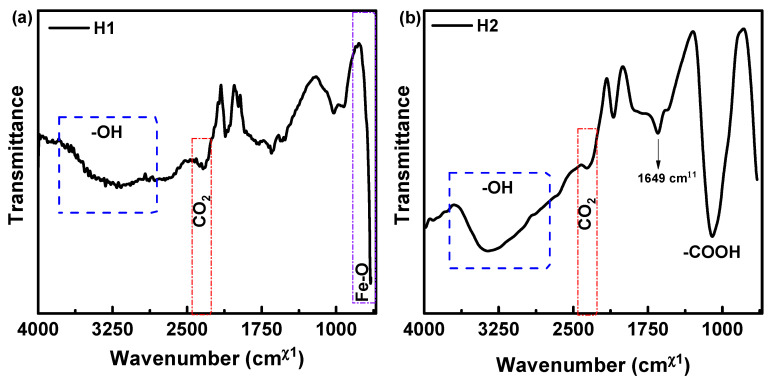
ATR-FTIR spectra for the (**a**) H1 and (**b**) H2 samples. The data were acquired in ATR mode but are presented in pseudo-transmittance coordinates (%T vs. wavenumber) for consistency with prior reports.

The system of iron-oxide NPs functionalized with quinoa extract was thermally treated to surpass the γ → α phase transition temperature (up 1173 K). At first glance, both spectra exhibit a similar overall profile, as depicted in Figure 4a,b. However, the most notable difference appears in Figure 4b around 1000 cm^−1^. A more detailed analysis reveals that the broad bands observed for both H1 (pure α-Fe_2_O_3_) and H2 (α-Fe_2_O_3_ functionalized with quinoa extract) at ~3300 cm^−1^ are associated with the -OH functional groups. Nevertheless, H2 displays narrower vibrational features, which result in an enhanced signal around ~2900 cm^−1^, likely attributable to phenolic compounds present in the quinoa extract [18,30]. The band near 2350 cm^−1^ corresponds to atmospheric CO_2_ [22].

For H1, the peak at 1650 cm^−1^ [31] may still be ascribed to the bending vibration of residual water molecules, whereas in the H2 ATR-FTIR spectrum, the Amide I group characteristic of quinoa proteins becomes more evident [32]. Finally, the most intense peak in H2, located at ~1000 cm^−1^, appears to originate from the contribution of C–N stretching vibrations [33] together with polymeric -COOH groups [18]. The results indicate that organic species from quinoa are still protecting the α-Fe_2_O_3_ NPs.

### 3.5. XPS Analysis

**Figure 5:**
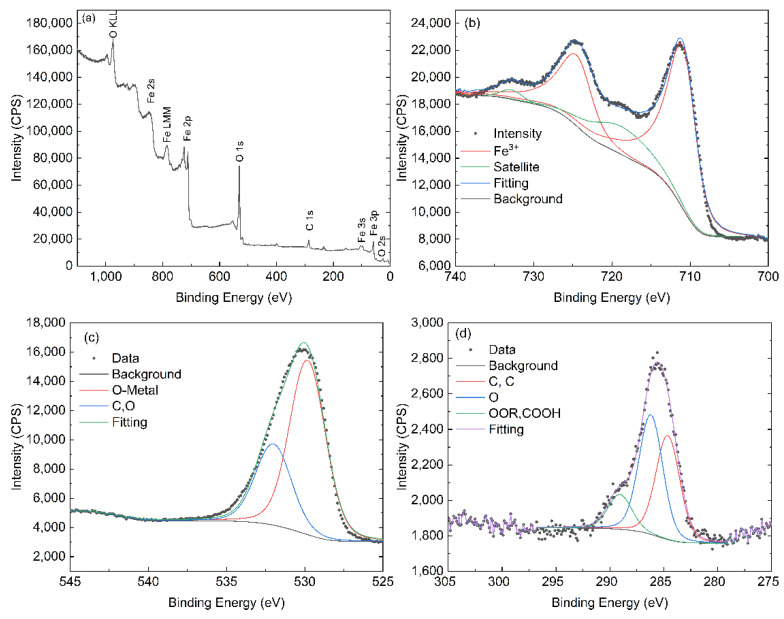
Survey (**a**), and high-resolution XPS spectra, and their respective fits for Fe 2p (**b**), O 1s (**c**), and C 1s (**d**) regions for the H1 sample.

**Figure 6:**
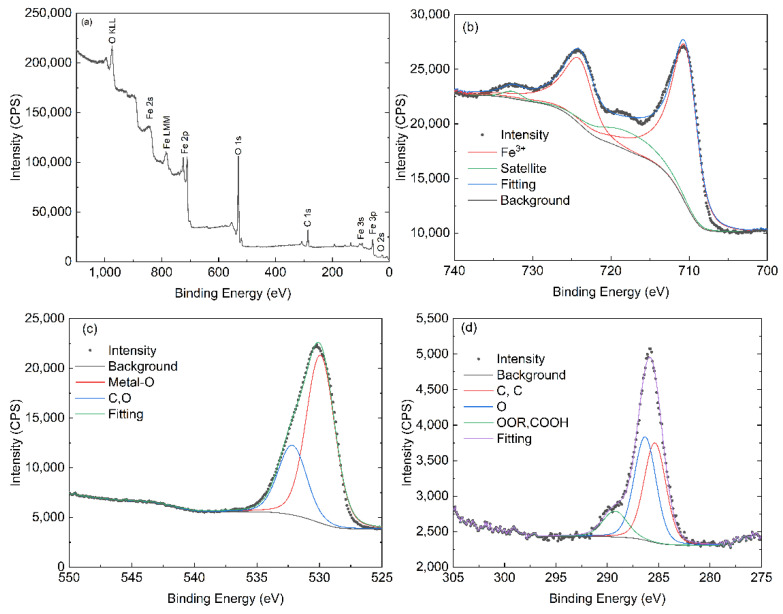
Survey (**a**), and high-resolution XPS spectra, and their respective fits for Fe 2p (**b**), O 1s (**c**), and C 1s (**d**) regions for the H2 sample.

XPS is a widely employed technique to probe the electronic structure and oxidation states of iron-oxide NPs, providing surface-sensitive insights that complement bulk analyses such as X-ray diffraction analysis. For instance, it should be pointed out that for an α-Fe_2_O_3_ crystal, the Fe 2p spectrum is dominated by (i) the Fe^3+^ oxidation state, typically characterized by a doublet (an Fe 2p_3_/_2_ main peak in the range of 709.9–711.0 eV binding energy (B.E.), and an Fe 2p_1_/_2_ feature at ~724.2–724.4 eV) and (ii) a distinct satellite peak near 718.9 eV, both are considered a fingerprint of Fe^3+^ [34,35,36]. The O 1s core level generally consists of two contributions: lattice oxygen (Fe–O) at 529.2–530.0 eV, and surface hydroxyl/adsorbed species at 530.9–531.4 eV [37,38,39]. Reported studies have also highlighted that slight shifts in binding energies or satellite positions can arise from particle size effects, surface hydroxylation, or crystallinity variations [40,41,42]. The XPS spectra for both samples are shown in Figure 5 and Figure 6, respectively.

The Fe 2p region was analyzed for both samples (Figure 5b and Figure 6b). It corroborates the presence of only the Fe^3+^ state in both samples. The Fe 2p_3/2_ peak located at ~710 eV B.E. agrees with the reported value for α-Fe_2_O_3_ or mixed overlapped α-Fe_2_O_3_ plus γ-Fe_2_O_3_ NPs [43]. But this differs from bulk α-Fe_2_O_3_ reported at 711 eV [4]. In addition, the H1 sample exhibits the Fe 2p_3_/_2_ peak at 710.4 eV, accompanied by a satellite feature at 718.7 eV, and an O 1s signal centered at 529.8 eV. These values are in excellent agreement with reported Fe^3+^ states in α-Fe_2_O_3_, and indicate that the surface of the NPs is dominated by lattice oxygen. Thus, the relatively high binding energy for Fe 2p_3_/_2_ and the strong satellite feature suggest a well-defined Fe^3+^ environment, consistent with a stoichiometric α-Fe_2_O_3_ phase.

The H2 sample, in contrast, displays the Fe 2p_3_/_2_ peak at 710.0 eV, and the satellite at 717.7 eV, with the O 1s peak at 529.9 eV. While the main Fe^3+^ feature is still within the expected range for α-Fe_2_O_3_, the ~0.4 eV downshift in binding energy and the ~1.0 eV downshift of the satellite peak relative to H1 are notable. These variations may be linked to subtle differences in the local chemical environment, such as enhanced electronic screening, surface hydroxylation, or a higher density of structural defects. Similar shifts have been reported for α-Fe_2_O_3_ NPs with reduced crystallinity or altered particle size, reflecting modifications in final-state relaxation processes [40,43].

The O 1s region for both samples depicted the presence of O-metal B.E. at 529.9 eV, likely associated with α-Fe_2_O_3_ or residual γ-Fe_2_O_3_ NPs still present in the H1 sample. In addition, the green shadow area corresponds to the organic content related to the atmospheric air commonly present after oxygen exposition of the samples. This is supported by the C 1s region, where the total organic content was 19% for H1 and 29% for H2, respectively. This last observation indicates that the H2 sample is more susceptible to physiosorb molecules from the air. This increment in the residual organic content can also be related to quinoa residues adsorbed on the α-Fe_2_O_3_ surface.

The XPS results for both samples are summarized in Table 3, where the binding energies of Fe 2p and O 1s are compared with literature values for α-Fe_2_O_3_. As shown, H1 closely matches the classical XPS signature of α-Fe_2_O_3_, with an Fe-2p_3_/_2_ peak at 710.4 eV, a satellite at 718.7 eV, and lattice oxygen at 529.8 eV. In contrast, H2 exhibits a lower Fe 2p_3_/_2_ binding energy (710.0 eV) and a satellite shifted to 717.7 eV, deviating slightly from the typical ~718.9 eV reported for Fe^3+^. This systematic downshift suggests subtle modifications in the electronic structure, which may originate from particle size effects, surface defects, or variations in crystallinity. Nevertheless, the O 1s peaks for both samples remain consistent with lattice oxygen, confirming the dominance of Fe–O bonds at the surface. Specifically, for both H1 and H2 samples, the O 1s signal is dominated by the lattice oxygen component at ~529.8–529.9 eV, with no significant indication of higher-binding-energy hydroxyl contributions. This suggests that the nanoparticle surfaces are relatively clean, although a more detailed deconvolution could reveal minor contributions from hydroxyl groups.

Moreover, the XPS survey spectra further corroborate the Fe–O stoichiometry of the samples. For H1, the relative atomic percentages were Fe = 15.2% and O = 54.9%, which approaches the ideal Fe:O ratio of 2:3 expected for stoichiometric α-Fe_2_O_3_. In contrast, H2 exhibited similar Fe content (20.5%) and higher oxygen contribution (60.6%), indicating a relative enrichment of oxygen due to organic environment at the surface in agreement with TGA. This oxygen excess, together with the slight downshift of the Fe 2p_3_/_2_ binding energy (710.0 eV vs. 710.4 eV in H1) and its satellite feature (717.7 eV vs. 718.7 eV), suggests subtle electronic modifications likely associated with surface hydroxylation, lattice defects, or structural disorder. Such differences in Fe/O ratio have been reported to strongly affect the surface chemistry and catalytic activity of α-Fe_2_O_3_ NPs, since oxygen-rich surfaces can stabilize additional –OH groups and modify the Fe^3+^ coordination environment. Therefore, the combined evidence from both peak positions and elemental ratios confirms that while both H1 and H2 are α-Fe_2_O_3_, H1 represents a closer-to-stoichiometric surface, whereas H2 is characterized by a more oxygen-rich and defect-sensitive surface chemistry.

Overall, H1 aligns closely with classical α-Fe_2_O_3_ XPS signatures, whereas H2 exhibits binding energy shifts, which are an indicative of subtle differences in surface or electronic structure. Thus, the comparative analysis reinforces that while both H1 and H2 samples are unequivocally α-Fe_2_O_3_, H2 presents surface or structural differences that distinguish it from the more stoichiometric H1. These findings emphasize the sensitivity of XPS to nanoscale variations and highlight the importance of correlating spectroscopic data with complementary structural and magnetic characterizations.

### 3.6. ^57^Fe Mössbauer Analysis of Nanohematites

**Figure 7:**
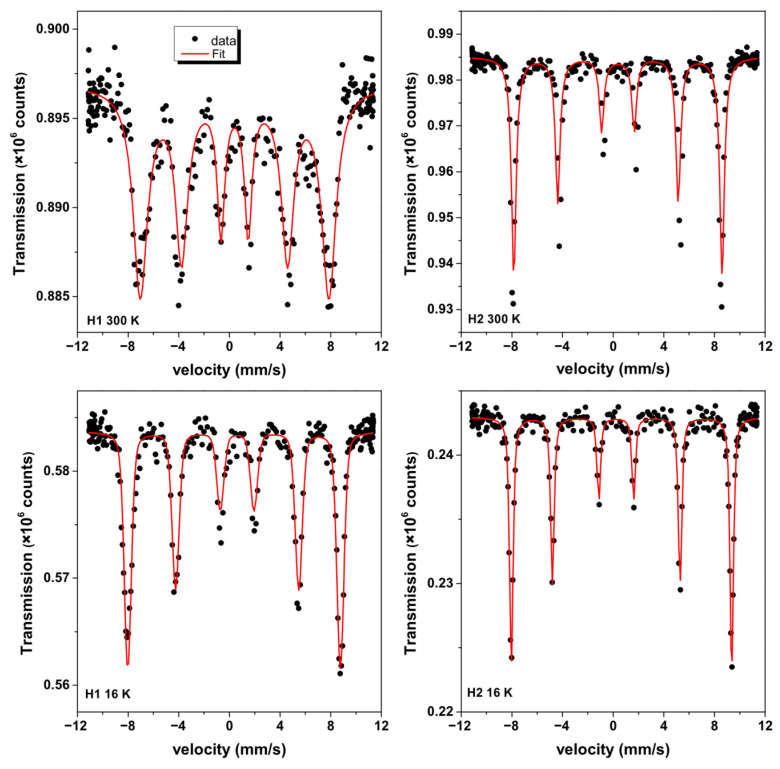
300 K (left-hand panel) and 16 K (right-hand panel) ^57^Fe Mössbauer spectra for the H1 and H2 samples.

Figure 7 depicts the 300 and 16 K ^57^Fe Mössbauer spectra for the H1 and H2 samples. At 300 K, the H1 sample has greater spin relaxation effect (broader absorption lines) than the H2 one, despite having close crystallites and mean particle nanosizes. Thus, Blume-Tjon relaxation patterns [44] were used to fit the H1 and H2 spectra. Table 4 summarizes the fitting hyperfine parameters. First, it must be noticed that the line width (LW) is almost double in the H1. The following explanations are given for the observed LW broadening. The H1 sample has a much broader distribution than the H2 sample and crystallites with smaller sizes, which is why these Fe^3+^ spin relaxation effects are observed. It also reinforces the presence of residual nanocrystallized γ-Fe_2_O_3_ NPs, with sizes < 10 nm in the H1 sample as also supported by TEM data. Thus, the existence of two magnetic phases also supports relaxation broadening effect of the H1 sample. On the other hand, the quinoa organic content in H2 helps to have a better crystallize α-Fe_2_O_3_ phase, as reinforced by the hyperfine parameter values at 16 K that are in good agreement with those found for bulk α-Fe_2_O_3_ [4]. However, the quadrupolar shifting ε of −0.24 mm/s for α-Fe_2_O_3_ NPs differs from the bulk value of 0.41 mm/s at 4.2 K [4], but goes in the same direction of the value reported by Bødker (−0.10 mm/s at 5 K) for weakly ferromagnetic 17 nm α-Fe_2_O_3_ NPs [8]. It is important to mention that the precedents of samples with magnetic nanometric coating (magnetic layers), undetected by X-ray diffraction, have been reported previously [45]. These magnetic layers cause surface magnetic disorder and interface coupling exchange phenomena.

### 3.7. VSM Analysis

**Figure 8:**
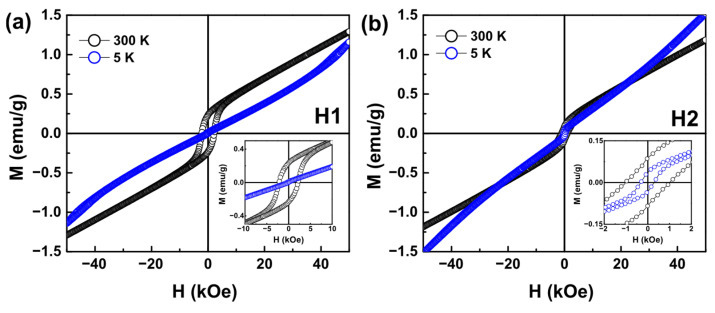
*M*(*H*) curves for the H1 (**a**) and H2 (**b**) samples measured at 300 K and 5 K. Insets display magnified views of the low-field region to highlight specific hysteresis features.

The 300 and 5 K *M*(*H*) curves are shown in Figure 8a,b for the H1 and H2 samples, respectively, and their main magnetic parameters are summarized in Table 5.

The magnetic characterization of the α-Fe_2_O_3_-based samples (H1 and H2) reveals a strong dependence on the thermal history and precursor nature. At 300 K, H1 exhibits a significantly higher coercivity (2.2 kOe) and remanence (0.23 emu/g) compared to those corresponding values for the H2 sample (1.0 kOe and 0.08 emu/g, respectively). On the other hand, it should be mentioned that the values of these parameters are unusually large for bulk-like α-Fe_2_O_3_, which behaves typically as weakly ferromagnet above the Morin transition due to the spin canting [4,46]. The enhancement in coercivity and remanence found in the H1 sample suggests the presence of residual nanoscale γ-Fe_2_O_3_ regions, where features like a higher *M*_s_ value (0.32 emu/g for H1 vs. 0.23 emu/g for H2) are a strong signature of a ferrimagnetic-like contribution probably due to the unconverted γ-Fe_2_O_3_ NPs (this result agrees with that obtained by Mössbauer spectroscopy). At 5 K, both samples exhibit a pronounced reduction in coercivity (0.3 kOe), a feature consistent with the freezing of surface spin dynamics and the weakening of anisotropy barriers at the nanoscale [47]. However, the much larger remanence retained in H1 at 5 K relative to H2 further indicates the persistence of a ferrimagnetic component (a secondary phase attributed to the γ-Fe_2_O_3_ NPs). All these magnetic results are supported by ^57^Mössbauer results. For example, at 300 K, the H1 sample displays a broad Mössbauer spectrum with marked spin relaxation effects, in sharp contrast with the narrow LW broadening observed for the H2 sample at the same temperature. The persistence of Mössbauer spectral broadening in the H1 sample, even at 16 K, suggests incomplete transformation of γ-Fe_2_O_3_ to α-Fe_2_O_3_ and the coexistence of small ferrimagnetic domains whose hyperfine fields are still distributed. By contrast, H2 evolves toward a spectrum characteristic of bulk α-Fe_2_O_3_ at 16 K, with well-defined sextets and a minimal spin relaxation effect. This distinction aligns with the magnetometry data, where H1 displays enhanced coercivity and remanence at 300 K, whereas H2 behaves more closely to single-phase α-Fe_2_O_3_.

Structural data further clarifies the origin of these differences. Both X-ray diffractograms indicated phase-pure α-Fe_2_O_3_ with average crystallite sizes of ~54–56 nm, yet TEM analysis of H1 revealed a bimodal particle size distribution (18.5 nm and 35.5 nm), while H2 showed a narrow distribution centered at ~28 nm. The bimodality in H1 likely reflects incomplete grain coarsening during the γ→α-Fe_2_O_3_ transformation, allowing for residual nanoscale ferrimagnetic domains. This scenario is consistent with the higher coercivity and Mössbauer spin relaxation observed. In contrast, the homogeneous size distribution of H2 facilitates a more complete structural and magnetic transformation into α-Fe_2_O_3_.

The differences are further explained by the nature of the precursors. H1 was derived from a 14 nm synthetic γ-Fe_2_O_3_ control subjected to thermal treatment under TG conditions, whereas H2 originated from the biosynthesized 7 nm γ-Fe_2_O_3_ NPs, produced with quinoa extract. The biosynthetic route not only generated smaller primary NPs, but may have promoted a more uniform thermal conversion to α-Fe_2_O_3_, resulting in fewer residual ferrimagnetic regions. Considering the magnetization of the parent phases (*M*_s_ = 69 emu/g for 14 nm control γ-Fe_2_O_3_ and *M*_s_ = 11 emu/g for the biosynthesized γ-Fe_2_O_3_), the amount of residual ferrimagnetic fraction required to explain the magnetic response of H1 is within the sub-percent range by mass. This explains why X-ray diffraction fails to detect the γ-Fe_2_O_3_ secondary phase, while Mössbauer relaxation and magnetometry remain sensitive probes of nanoscale heterogeneity.

In summary, the combined evidence demonstrates that the H1 sample retains a minor fraction of residual γ-Fe_2_O_3_, which significantly enhances its coercivity and remanence, whereas H2 represents a nearly complete conversion to α-Fe_2_O_3_. The contrasting behavior emphasizes the critical role of precursor type, particle size distribution, and thermal pathway in determining the final magnetic properties of α-Fe_2_O_3_ NPs. These results highlight the necessity of combining structural and morphological (X-ray diffraction, TEM) and local probes (^57^Mössbauer spectroscopy) with magnetometry to unravel subtle phase coexistence in iron-oxide phases.

## 4. Conclusions

This work demonstrates that hematite NPs, obtained from heating under controlled conditions nanomaghemite precursors, exhibit distinct structural and magnetic behaviors, features that are strongly influenced by the history of precursors. Although X-ray diffraction confirmed phase-pure α-Fe_2_O_3_ for both H1 (obtained from heating the synthetic precursor, 14 nm γ-Fe_2_O_3_) and H2 (obtained from heating the biosynthesized precursor, 7 nm γ-Fe_2_O_3_ with quinoa extract), complementary probes revealed critical differences. For H1, TEM revealed a bimodal size distribution, ^57^Mössbauer spectra showed persistent spin relaxation effects even at low temperatures (16 K), and magnetometry indicated enhanced coercivity and remanence. Importantly, XPS detected Fe^3+^ as the dominant state but also revealed subtle satellite features, consistent with residual γ-Fe_2_O_3_ or surface disorder. These findings indicate incomplete transformation and the retention of magnetically active ferrimagnetic fractions. On the other hand, the H2 samples showed the following features: TEM indicated uniform particles (~28 nm), ^57^Mössbauer spectra (of course, hyperfine parameters) closely matched with those values found in bulk α-Fe_2_O_3_, and magnetometry showed lower coercivity and remanence. XPS confirmed Fe^3+^ without additional features, supporting a more complete γ to α-Fe_2_O_3_ transformation. Together, the applied multi-technique approach demonstrates that while both samples crystallized as α-Fe_2_O_3_, only H2 achieved a homogeneous phase, whereas H1 retained nanoscale ferrimagnetic residues detectable by XPS and ^57^Mössbauer. These results emphasize that biosynthetic functionalization enables more complete γ → α-Fe_2_O_3_ phase transformation, consequently, leading to α-Fe_2_O_3_ NPs with tailored and reproducible magnetic behavior for environmental and technological applications.

## Figures and Tables

**Table 1:** Rietveld refinement parameters, statistical parameters, and crystallite sizes of the H1 and H2 samples. R_p_ (%), R_wp_ (%), and R_exp_ (%) are the profile residual, the weighted profile residual, and the expected profile residual, respectively, while χ^2^ is the goodness of the refinement.

Sample	Phase	Caglioti Parameters			Lattice Parameters (Å)	*D*_Rietveld_ (nm)	Weight Fraction (%)	Statistical Parameters
		U	V	W				
H1	α-Fe_2_O_3_	0.011	−0.055	0.058	a = 5.034(1) b = 5.034(1) c = 13.745(1) α = β = 90° γ = 120°	54(1)	100	R_p_ = 74.2% R_wp_ = 34.1% R_exp_ = 19.9% χ^2^ = 2.94
H2	α-Fe_2_O_3_	0.021	−0.062	0.057	a = 5.031(1) b = 5.031(1) c = 13.737(1) α = β = 90° γ = 120°	56(1)	100	R_p_ = 73.8% R_wp_ = 34.0% R_exp_ = 19.9% χ^2^ = 2.91

**Table 2:** Representative Miller planes and interplanar distances for the H1 and H2 samples.

**H1**			
**Miller Plane**	**2*θ* (°)**	***d*** **(nm)**	***d*** **_ref_** **. (nm)**
(104)	33.17	0.2700	0.2703
(110)	35.68	0.2517	0.2519
**H2**			
**Miller Plane**	**2*θ* (°)**	***d*** **(nm)**	***d*** **_ref_** **. (nm)**
(104)	33.19	0.2702	0.2703
(110)	35.67	0.2516	0.2519

**Table 3:** Binding energies (eV) for Fe 2p_3/2_, Satellite, and O 1s components found in the literature and in the H1 and H2 samples.

Sample/Reference	Fe 2p_3_/_2_ (eV)	Satellite (eV)	O 1s (eV)	Notes
Literature (α-Fe_2_O_3_, Fe^3+^) [34,35,36]	709.9–711.0	~718.9	529.2–530.0 (lattice O); 530.9–531.4 (–OH)	Classical α-Fe_2_O_3_ XPS signature
H1 (this work)	710.4	718.7	529.8	Very close to classical α-Fe_2_O_3_; Fe^3+^ well defined; lattice oxygen dominant
H2 (this work)	710.0	717.7	529.9	Fe^3+^ state preserved, but satellite shifted ~1 eV lower; suggests subtle changes in surface/electronic environment

**Table 4:** ^57^Fe Mössbauer hyperfine parameters obtained from the fittings of the H1 and H2 spectra at 300 and 16 K: IS is the isomer shift (mm/s), *B*_hf_ corresponds to the hyperfine magnetic field (T), ε is the quadrupole shifting (mm/s), LW indicates the linewidth (mm/s), and R.A.A. corresponds to the relative absorption area of each component (%).

Sample	IS (mm/s) ±0.01	*B*_hf_ (T) ±0.5	*ε* (mm/s) ±0.01	LW (mm/s) ±0.01	R.A.A. (%) ±1
H1-300 K	0.41	47.5	0.00	0.63	100
H1-16 K	0.49	52.2	−0.24	0.54	100
H2-300 K	0.38	51.1	−0.19	0.35	100
H2-16 K	0.46	54.1	0.42	0.18	100

**Table 5:** Magnetic parameters (coercivity *H*_c_, saturation magnetization *M*_s_, and remanent magnetization *M*_r_) of the H1 and H2 samples were measured at 300 K and 5 K using VSM.

Sample	T	*H*_c_	*M*_s_	*M*_r_
	(K)	±0.1 (kOe)	±0.01 (emu/g)	±0.01 (emu/g)
H1	300	2.2	0.32	0.23
	5	0.3	-	-
H2	300	1.0	0.23	0.08
	5	0.3	-	0.03

## Data Availability

The original contributions presented in this study are included in the article. Further inquiries can be directed to the corresponding author.
